# Macrophages and small extracellular vesicle mediated-intracellular communication in the peritoneal microenvironment: Impact on endometriosis development

**DOI:** 10.3389/frph.2023.1130849

**Published:** 2023-04-03

**Authors:** Yifan Wang, Rebecca A. Dragovic, Erin Greaves, Christian M. Becker, Jennifer H. Southcombe

**Affiliations:** ^1^ Nuffield Department of Women's and Reproductive Health, Oxford Endometriosis CaRe Centre, Nuffield University of Oxford, Oxford, United Kingdom; ^2^ Division of Biomedical Sciences, Warwick Medical School, University of Warwick, Coventry, United Kingdom

**Keywords:** endometriosis, small extracellular vesicle, peritoneal microenvironment, endometriotic stromal cell, macrophage

## Abstract

Endometriosis is an inflammatory disease that is defined as the growth of endometrium-like tissue outside the uterus, commonly on the lining of the pelvic cavity, visceral organs and in the ovaries. It affects around 190 million women of reproductive age worldwide and is associated with chronic pelvic pain and infertility, which greatly impairs health-related life quality. The symptoms of the disease are variable, this combined with a lack of diagnostic biomarkers and necessity of surgical visualisation to confirm disease, the prognosis can take an average timespan of 6–8 years. Accurate non-invasive diagnostic tests and the identification of effective therapeutic targets are essential for disease management. To achieve this, one of the priorities is to define the underlying pathophysiological mechanisms that contribute to endometriosis. Recently, immune dysregulation in the peritoneal cavity has been linked to endometriosis progression. Macrophages account for over 50% of immune cells in the peritoneal fluid and are critical for lesion growth, angiogenesis, innervation and immune regulation. Apart from the secretion of soluble factors like cytokines and chemokines, macrophages can communicate with other cells and prime disease microenvironments, such as the tumour microenvironment, *via* the secretion of small extracellular vesicles (sEVs). The sEV-mediated intracellular communication pathways between macrophages and other cells within the peritoneal microenvironment in endometriosis remain unclear. Here, we give an overview of peritoneal macrophage (pM*Φ*) phenotypes in endometriosis and discuss the role of sEVs in the intracellular communication within disease microenvironments and the impact they may have on endometriosis progression.

## Introduction

Endometriosis is a chronic inflammatory disease that affects approximately 10% of women of reproductive age worldwide (1). It is characterized as the ectopic growth of endometrium-like tissue, most commonly along the mesothelial cell layer lining the peritoneal cavity, but also in the form of ovarian endometriosis cysts (endometrioma) or below the peritoneal surface as deep nodules (2). Clinical symptoms include cyclical and non-cyclical pelvic pain, dysmenorrhea, and pain during and after sexual intercourse, defecation and emptying the bladder (2). Around 30% to 50% of patients with endometriosis present with subfertility (3).

Depending on the location, depth and size of lesions and adhesions, endometriosis can be divided into stages I-IV using the *r*ASRM (revised American Society for Reproductive Medicine) classification system (4); deeply infiltrating endometriosis can be further classified following the ENZIAN criteria (5) and pregnancy outcomes can be predicted using the Endometriosis Fertility Index (EFI) (6). With the improvement of medical technologies, imaging tools (MRI and ultrasound) have shown reasonable specificity and sensitivity to aid diagnosis of endometrioma and deep endometriosis (7). The definitive diagnosis of endometriosis, especially peritoneal endometriosis, still requires laparoscopy (1).

As an oestrogen-driven chronic inflammatory disease, endometriosis primarily affects women during reproductive age. Clinically, it often manifests itself as early as adolescence (1). Dependent on geographical locations and accessibility of health care, there exists a delay of 6–8 years between the onset of symptoms and diagnosis (8). Shortening this gap requires increased awareness both in the general population and in the medical community, improvement of positive and negative predicative value of current imaging modalities particularly for peritoneal endometriosis and development of clinically reliable biomarkers. Furthermore, both medical and surgical approaches are associated with high recurrence rates and significant side effects (9, 10). For many patients, therefore, the disease generates long-term impairment to their quality of life, and consequently it is a substantial burden to healthcare systems and within society (1).

Retrograde menstruation is the most widely accepted theory implicated in the aetiology of endometriosis (11). This theory proposes that endometriosis lesions develop from endometrial cells and tissue flowing backward from the uterine cavity during menses, *via* the Fallopian tubes, into the peritoneal cavity (11). However, other mechanisms involved in the regulation of cell adhesion and proliferation must exist, as this retrograde menstruation occurs in as many as 90% of females (12). Endometriosis lesion architecture is variable but is usually composed of endometrial stromal and epithelial cells, with immune cell infiltration, fibrogenesis, neovascularisation, and innervation (2, 13). Endometrial stromal cells are the most predominant cell population in ectopic lesions and are thought to be mostly responsible for lesion attachment to the peritoneum (14).

### Immune dysfunction in the peritoneal microenvironment of endometriosis

Endometriotic lesions and the mesothelial cell layer are exposed to immune cells in the peritoneal fluid (PF). Mass cytometry (15) and single-cell RNA sequencing analysis (16) has revealed distinct immune cell profiles of PF between endometriosis patients and controls. Cellular profiling studies identified over 40 types of immune cells in the PF, including monocytes and macrophages (the most abundant cell population), natural killer (NK) cells and neutrophils from the innate immune system, as well as T and B cells from the adaptive immune system (15). Recent evidence has suggested that peritoneal immune dysregulation facilitates the growth of endometriotic lesions (17). For example, decreased NK cell cytotoxicity was observed in PF of women with endometriosis compared to control women (17). The T helper (CD4^+^) immune pattern in PF of endometriosis patients is shifted toward a Th2 anti-inflammatory immune response favouring lesion growth (18, 19). Recently regulatory T (Treg) cells have also been implicated in disease development, through interactions with endometrial stromal cells and macrophages (20–22).

### The role of peritoneal macrophages in endometriosis

Macrophages are the most abundant immune cell population in PF, accounting for almost 50% (15). In addition to tissue-resident macrophages, monocyte-derived macrophages are recruited to the peritoneal cavity when local inflammation occurs (23). Elevated numbers of macrophage are found in PF of endometriosis patients (15). These peritoneal macrophages (pM*Φ*) are recruited and get activated under the influence of macrophage growth factors and chemokines, such as colony-stimulating factor-1 (CSF-1) and monocyte chemoattractant protein-1 (MCP-1/CCL2) (24, 25). Mesothelium, endometriotic stromal cells and nerve fibres participate in the chemotactic recruitment of macrophages to the pelvic cavity in an oestrogen-dependent manner (26, 27). Oestrogen acts on pM*Φ* and endometriotic lesions *via* the oestrogen receptors alpha (ERα) and beta (ERβ) (28, 29).

The activated pM*Φ* exhibit both pro-inflammatory and pro-repair phenotypes (15). They produce numerous cytokines and growth factors in the peritoneal microenvironment, such as interleukin-1 beta (IL-1β), interleukin-6 (IL-6), interleukin-8 (IL-8), interleukin-12 (IL-12), tumour necrosis factor-alpha (TNF-α), vascular epithelial growth factor (VEGF) and transforming growth factor-beta 1 (TGF-β1) to induce endometriosis lesion implantation, growth and angiogenesis (2, 17, 30). Notably, Treg cells can promote macrophage polarization with pro-repair phenotypes *via* the secretion of soluble fibrinogen-like protein 2 (22). In addition, pM*Φ* from endometriosis patients have impaired phagocytotic abilities caused by the downregulation of CD36 (31, 32). These may contribute to the survival and attachment of refluxed endometrial cells and tissue. Furthermore, increased pM*Φ* abundance is correlated with pelvic pain scores in endometriosis patients (33), but the severity of pain symptoms does not correlate with rASRM stages, suggesting complex mechanisms (34). Inflammatory responses in endometriosis modulate pain by activating and sensitising peripheral nerve fibres, and long-term peripheral nociceptive input leads to central sensitisation (35, 36). Macrophages are attracted to nerve fibres under the influence of CSF-1 and CCL-2 (27, 37), and the recruited macrophages secrete nerve growth factors such as insulin growth factor 1, and VEGF, promote neurogenesis and nerve sensitization mediated by oestrogen (29, 38).

**Figure 1:**
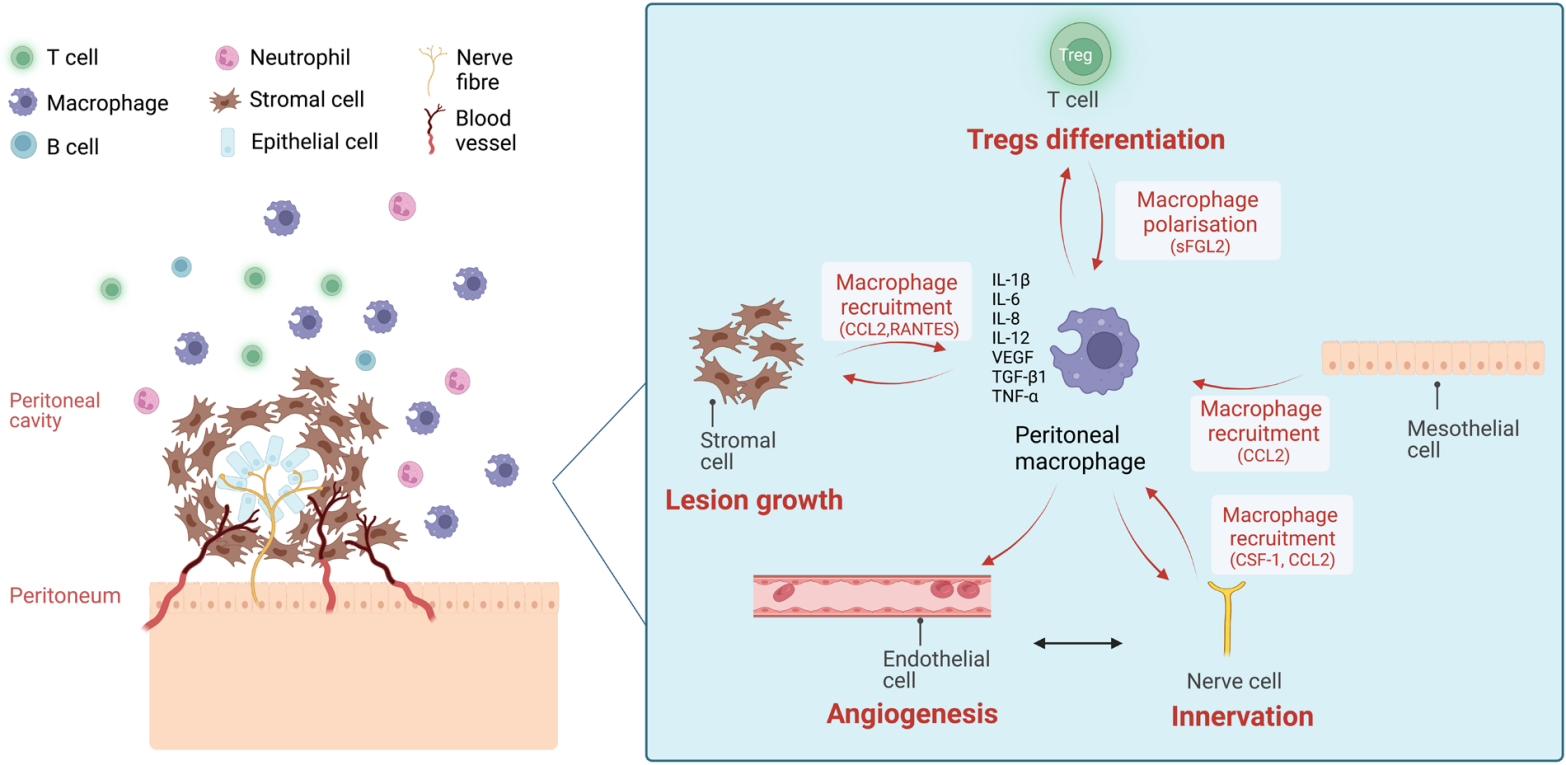
The chemokine-mediated intercellular communication of macrophages and other cells in the peritoneal microenvironment of endometriosis. Ectopic endometriotic lesions in the peritoneal cavity are composed of stromal cells and epithelial cells and infiltrated with blood vessels and nerve fibres. In the surrounding peritoneal fluid (PF), immune cells are present, including macrophages, neutrophils, T cells and B cells. The number of total peritoneal macrophages and CD206^+^/CD163^+^ macrophages are increased in PF of endometriosis patients. Stromal cells from the endometriotic lesions, mesothelial cells from peritoneum, nerve fibres attract macrophages to peritoneal cavity *via* the secretion of attractant factors like monocyte chemoattractant protein-1 (MCP-1/CCL2), colony-stimulating factor-1 (CSF-1), and RANTES (CCL5). Existing peritoneal macrophages also secrete MCP-1 to attract more macrophages. Treg cells induce macrophage polarisation into pro-repair subtypes *via* the secretion of soluble fibrinogen-like protein 2 (sFGL2), favouring lesion growth. On the other hand, peritoneal macrophages facilitate lesion growth, angiogenesis, neurogenesis and Treg differentiation *via* the secretion of cytokines and growth factors including interleukin-1β (IL-1β), interleukin-6 (IL-6), interleukin-8 (IL-8), interleukin-12 (IL-12), tumour necrosis factor alpha (TNF-α), vascular epithelial growth factor (VEGF) and transforming growth factor beta 1 (TGF-β1). (Created with biorender.com).

These studies show that pM*Φ* are associated with endometriosis progression through intercellular communication with other cells in peritoneal microenvironments (Figure 1). Intercellular crosstalk is not only limited to soluble factors; small extracellular vesicles (sEVs) also mediate cell communication. sEVs, previously referred to as exosomes, are nanosized lipid-bilayer vesicles (30 nm to 150 nm) released by cells (39), which are present in almost all biological fluids, including PF (40). They are formed by the inward budding of multivesicular endosomes (MVEs) and secreted after the fusing of MVEs with the cell surface (39). sEVs are enriched in specific cargoes (proteins, lipids, nucleic acids and metabolites), reflecting their cell of origin, and they deliver these to recipient cells to modulate their activities (41). Noticeably, sEVs are elevated in several diseases where they display altered phenotypes (39). Studies characterising the role of sEVs have advanced our knowledge of the pathology of various diseases, including cardiovascular diseases (42), neurological diseases (43), autoimmune disorders (44) and cancer (45). It has become clear that sEVs are important mediators of intracellular communication in disease microenvironments and they have emerged as valuable biomarkers and potential therapeutic targets (46, 47).

In this review, we will discuss the phenotypes of pM*Φ* and sEV-mediated intracellular crosstalk in several different disease microenvironments, and consider how these may contribute to endometriosis development.

## Peritoneal macrophage phenotypes in endometriosis

pM*Φ* comprise a heterogenous population of immune cells originating from various locations (23). They are highly plastic cells that can differentiate into specific subtypes in response to local stimuli. Different subtypes of tissue-resident macrophages present various receptors, secrete distinct chemokines and cytokines, and consequently play specific functional roles (48). Flow cytometry studies have reported the heterogeneity within human pM*Φ* populations and various subsets are identified by assessing the expression of canonical markers, such as CD14, CD16 and HLA-DR (49) and distinguished by expression of Complement Receptor of the Immunoglobulin subfamily (CRIg) and CCR2 (50). In endometriosis patients, one study identified two subpopulations of pM*Φ* as HLA-DR^+^ CD14^lo^ and HLA-DR^+^ CD14^hi^ (33) and another study revealed two populations of pM*Φ* based on the expression of CD14 and CD68 (51). Recently, single cell RNA-Seq analysis identified seven distinct subtypes of macrophages in PF from an endometriosis patient (16) and reported five subpopulations of tissue resident and blood infiltrated macrophages in ectopic lesions (52). Validation of the characterisation and functional studies of these subtypes are now required to determine if the newly identified pM*Φ* subtypes have distinct roles in endometriosis progression.

In mouse models of experimental endometriosis, the origins and phenotypes of pM*Φ* have been thoroughly investigated. Tissue-resident macrophages are seeded from the embryo yolk sac and foetal liver, and monocyte-derived macrophages are seeded from the bone marrow during adulthood (23). In mice, pM*Φ* are characterised into large peritoneal macrophages (LpM; F4/80^hi^ MHC II^lo^) and monocyte-derived small peritoneal macrophages (SpM; F4/80^lo^ MHC II^hi^) (23). LpM are dominant in the peritoneal cavity and consist primarily of self-renewing embryonic-derived cells (53). They perform immunosurveillance in the peritoneal cavity (54). Under inflammatory conditions like endometriosis, monocytes infiltrate into pelvic cavity and transform into pro-inflammatory SpM, which eventually differentiate into LpM (55). LpM, therefore, consists of embryonic-derived and monocyte-derived cells (55). Notably, the tissue-resident, embryonic-derived LpM promote lesion growth, while monocyte-derived LpM appear to limit the growth of lesions in an endometriosis mouse model (56). These findings on the origins of pM*Φ* and SpM/LpM functions in the mouse model, cannot be directly translated to the human macrophage system, where pM*Φ* display a higher level of complexity and heterogeneity. Further research is critically required on the origins, phenotypes and functions of human pM*Φ* in homeostasis and disease states.

## Small extracellular vesicle-mediated crosstalk in tissue-specific microenvironments

### The uptake and functions of sEVs

sEVs are important cellular communicators in both physiological and pathological processes, through transferring functional proteins, lipids, and nucleic acids to recipient cells (39). While the cargo in sEVs generally reflects that of the parent cells, the RNA in sEVs tends to consist of small noncoding RNAs, like micro RNAs (miRNAs) and RNA fragments (57, 58). Altered miRNA expression profiles are observed in endometriosis patients and as miRNAs also function as epigenetic machinary, sEVs could contribute to the process (59). The uptake of sEVs can be local to the site of release or distant as they circulate in biological fluids (60). Some sEVs can be exclusively taken up by certain cell types, for example in the case of sEV mediated organ-specific metastasis in cancer (61). Cellular uptake is mediated by the surface composition of the vesicles (62). Once sEVs reach the recipient cells, they can either trigger signalling by directly interacting with surface receptors, fusing with the plasma membrane, or be internalised (60). For the functional use of sEV-encapsulated miRNAs and RNAs, sEVs need to get internalised, bypass degradation and release cargos targeted to endoplasmic reticulum for translation (63).

### sEV-mediated crosstalk in healthy and diseased microenvironments

Endometriotic lesions share some clinical similarities with cancer. For instance, both exhibit a metastatic phenotype with adhesion, invasion and neuroangiogenesis, although the lack of driver mutations limit the malignant potential of most forms of endometriosis (2). There are a wealth of studies showing that tumour-associated macrophage (TAM) derived-sEVs (TAM-sEVs) interact with each other and other cells to promote tumour progression. TAMs are one of the most studied disease-associated macrophage populations. TAM-sEVs have been found to regulate tumourigenesis (64), metastasis (65, 66) and drug resistance (67), by transferring miRNAs and proteins to other cells in various tumour microenvironments. The regulations conferred are likely specific and dynamic to the cancer type. For example, tumour-derived sEV miR-934 induces macrophage polarisation into anti-inflammatory subtypes, promoting liver metastasis of colorectal cancer (68). Notably, the sEV-mediated regulation of TAMs is not limited to inducing anti-inflammatory polarisation. In oral squamous cell carcinoma, tumour-derived sEVs can activate pro-inflammatory TAMs to promote tumour migration (69). In addition, melanoma cell-derived sEVs can transform lipopolysaccharide (LPS) and interferon-gamma (IFN-*γ*) stimulated macrophages to pro-inflammatory and pro-angiogenic TAMs, which present strong differences in gene expression compared to macrophages stimulated by interleukin-14 (IL-4) + interleukin-13 (IL-13) and LPS + IFN- *γ*, and higher survival rates (70). These studies highlight the heterogeneity of TAMs and their complex roles in tumour microenvironments.

Importantly, sEV-mediated communication between TAMs and other cells within the tumour microenvironment is bilateral. For example, in gastric cancer, TAMs promote the migration of gastric cancer cells by transfer of functional Apolipoprotein E *via* sEVs to activate the PI3K-Akt signalling pathway (65). TAM-sEVs do not only directly regulate cancer cells—in cases of pancreatic ductal adenocarcinoma TAM-sEVs carrying miR-155–5p and miR-211–5p promote angiogenesis and tumour growth by suppressing E2F2 expression in endothelial cells (71). In epithelial ovarian cancer TAM-sEVs induce Treg/T helper 17 cell imbalance, contributing to tumour progression and metastasis (72). In summary, in the tumour microenvironment, TAMs can promote tumorigenesis and metastasis by directly regulating tumour cells, or by indirectly targeting endothelial and immune cells, *via* sEVs.

It is important to remember that sEVs also influence eutopic endometrium function in both physiological and pathological processes. Protein cargos of sEVs derived from endometrial epithelial cells enhance the adhesive capacity of trophoblast, potentially contributing to embryo implantation (73). Intriguingly, in patients with adenomyosis (a condition where endometrial-like cells grow into the myometrium causing heavy menstrual bleeding, pain and infertility, often in association with endometriosis) (74), sEVs secreted by endometrial organoids contain miRNAs associated with pregnancy complications and adenomyosis progression (75).

### The sEV-mediated crosstalk in the peritoneal microenvironment of endometriosis

These findings from cancer and endometrium studies raise the possibility that macrophage-derived and endometriosis tissue-specific sEVs could have an impact on endometriosis progression through concerted cell targeting in the lesion microenvironment (76–78). Indeed, similar to TAM, pM*Φ* are also regulated by sEV mediated signalling networks. In an endometriosis mouse model, sEVs derived from stromal cells induced macrophage polarisation into an anti-inflammatory subtype with decreased phagocytotic abilities, leading to increased lesion size (79). One recent study found that ectopic stromal cells collected from recurrent ovarian endometriosis patients induced anti-inflammatory polarisation of macrophages *via* the secretion of sEV-derived Legumain pseudogene 1 (EV-LGMNP1), a newly identified pseudogene of LGMN (80). LGMN is highly expressed in many cancers and appears to promote cancer progression (81, 82). Intriguingly in the following retrospective clinical cohort study (*n* = 73) a higher serum EV-LGMNP was detected in recurrent endometriosis patients (80).

pM*Φ*-sEVs, on the other hand, have been shown to transfer miR-22–3p to endometrial stromal cells, enhancing cell proliferation, migration, and invasion through the regulation of the SIRT1/NF-*κ*B signalling pathway (83). Another study revealed that pM*Φ*-sEVs induce proliferation and migration of ectopic stromal cells *in vitro* and promote lesion growth in an endometriosis mouse model *via* the transfer of the long non-coding RNA (lncRNA) CHL1-AS1 (84). lncRNA CHL1-AS1 is the antisense of the *CHL1* gene, which can suppress or promote cancer development at different stages (85). Overexpression of the *CHL1* gene and lncRNA CHL1-AS1 has been found in the ectopic endometrium from ovarian endometriosis patients (86). Interestingly, sEVs from LPS-induced macrophages can reduce endometriosis lesion growth by repolarising anti-inflammatory macrophages into pro-inflammatory subtypes in mice (87). These macrophage-derived sEVs also repress stromal cell migration and angiogenesis *in vitro* (87). These above-mentioned studies suggest that macrophage-derived sEVs could target various cells and pathways in endometriosis. Accordingly, these sEVs may promote or supress endometriosis progression, determined by the phenotypes of the macrophages that they are derived from. The majority of the literature has focussed on pM*Φ*-sEVs and, to date, there exists a lack of data on the role of lesion-resident macrophage-derived sEVs.

An additional source of sEVs relevant to endometriosis are those from stromal cells. Apart from regulating pM*Φ*, sEVs derived from endometrial stromal cells are found to induce neuroangiogenesis (88). Additionally, sEVs from endometrial stromal cells from endometriosis patients exhibit differential profiles of miR-21 and lncRNA antisense hypoxia inducible factor (aHIF), promoting proangiogenic properties of endothelial cells (89, 90). lncRNA aHIF derived from endometrial stromal cells target VEGF, a strong pro-angiogenic molecule, which is highly expressed in endometriosis lesions and PF of endometriosis patients (90). Of note, sEV shuttled miR-21, has been linked to tumour progression through targeting of cancer cells, endothelial cells and immune cells (like macrophages) as an apoptosis suppressor (91). Interestingly, stromal cell sEV secreted miR-214 and miR-214–3p are found to supress fibrosis of endometriosis lesions in murine models (92, 93); miR-214–3p was significantly downregulated and its target, connective tissue growth factor, is upregulated in ectopic lesions from endometriosis patients (93). Together, the evidence suggests endometriosis-specific stromal derived sEVs may contribute to disease progression. Recently, miR-30c encapsulated in sEVs derived from endometriotic epithelial cells was found to supress epithelial cell invasion and migration and attenuate endometriosis progression in a mouse model (94).

These *in vitro* and *in vivo* studies indicate that the peritoneal microenvironment will likely contain a variety of sEVs, and that these sEVs may carry key factors instrumental in the pathogenesis of endometriosis. Combined with the known miRNA and lncRNA, a distinct sEV protein profile has been identified in a mass spectroscopy proteomic study of PF-derived sEVs from endometriosis patients, compared to controls (39). Five proteins, peroxiredoxin-1, histone H2A type-2-C, annexin A2, inter-α-trypsin inhibitor heavy chain H4 and tubulin alpha-chain were exclusively present in sEVs in the PF from women with endometriosis (39). One of the proteins, Annexin A2 has been found to be highly expressed in ectopic stromal cell-derived sEVs, and promotes angiogenesis and stromal cell proliferation and migration by activating ERK1/2 STAT3 pathways (95). Future work is required to build on these data and establish key pathways that could be targeted for therapies, or function as biomarkers for disease severity.

## Future perspectives

**Figure 2:**
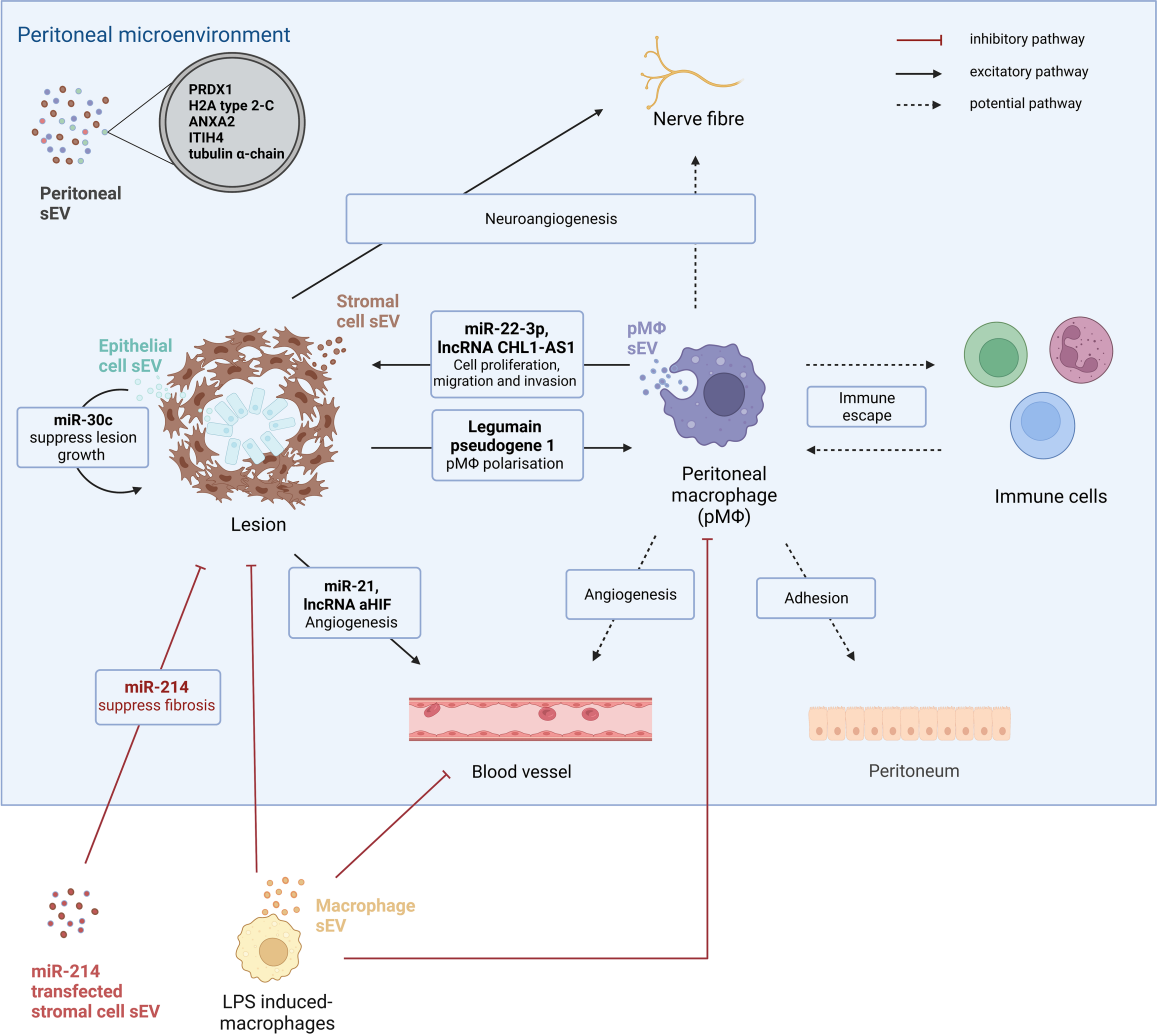
The small extracellular-mediated intracellular communication in endometriosis. Small extracellular vesicles (sEVs) are present in peritoneal fluid and distinct protein profiles are shown in peritoneal sEVs from endometriosis patients compared to controls. Five proteins, peroxiredoxin-1 (PRDX1), histone H2A (H2A) type-2-C, annexin A2 (ANXA2), inter-α-trypsin inhibitor heavy chain H4 (ITIH4) and tubulin alpha-chain are solely present in peritoneal sEVs from endometriosis patients. sEVs are important intracellular communicators between different types of cells in the peritoneal microenvironment of endometriosis. One study has revealed that stromal cell-derived sEVs regulate macrophage polarisation by delivering legumain pseudogene 1 and promote angiogenesis *via* miR-21 and lncRNA aHIF. Additionally, stromal cell-derived sEVs promote neuro-angiogenesis. Endometrial epithelial cell sEVs inhibit lesion growth by transferring miR-30c. Peritoneal macrophages also secrete sEVs. pM*Φ* -derived sEVs promote lesion growth by delivering miR-22-3p and lncRNA CHL1-AS1 to stromal cells. pM*Φ* could potentially regulate angiogenesis, neuroangiogenesis and immune escape in endometriosis progression *via* sEVs. As a future potential therapeutic approach, miR-24 transfected sEVs from stromal cells are found to supress fibrosis in an endometriosis mouse model. LPS induced-macrophage-derived sEVs could attenuate endometriosis progression by repolarising pM*Φ*, inhibiting angiogenesis and stromal cell proliferation.

Macrophages play a central role in endometriosis establishment and progression and exhibit a high level of heterogeneity. Recent studies have identified various human pM*Φ* subpopulations using different experimental approaches. pM*Φ* achieve bilateral interactions with a broad range of cells in endometriosis (Figure 1). Along with soluble factors, pM*Φ* will likely secrete sEVs with functional moieties, and the pM*Φ* themselves are likely affected by sEVs from the microenvironment, akin to tumour microenvironments (Figure 2). Recent studies have revealed the significance of stromal cell-derived sEVs in several aspects of endometriosis progression including angiogenesis, neuroangiogenesis and macrophage polarisation by transferring RNAs and proteins. The research on macrophage-derived sEVs, on the other hand, is limited to the regulation of pM*Φ*-sEVs on cell proliferation, migration and invasion of stromal cells. We propose a succession of pathways in which pM*Φ*-sEVs may be involved in endometriosis: when ectopic endometrial tissue and cells enter the abdominal cavity during menstruation, pM*Φ*-sEVs regulate other immune cells (e.g.,: T cells) impairing immune surveillance, facilitating lesion implantation to peritoneal surfaces. Once the lesions are attached, the endometrial lesions will further release sEVs to transform pM*Φ* into lesion-favouring subtypes. sEVs secreted from these macrophages will then communicate with other immune cells, endothelial and nerve cells to sustain immune evasion, promote lesion proliferation, and induce fibrosis, angiogenesis and neurogenesis (38). Future work to investigate sEVs derived from endometriosis associated-macrophages including lesion-resident macrophages are critically required.

One pivotal point when conducting sEVs characterisation studies in endometriosis is that many cellular and intracellular activities in the peritoneal microenvironment are oestrogen dependent (1, 29). Ectopic endometriotic lesions contain oestrogen receptors and enzymes such as P_450−_aromatase to convert androgens into potent 17β-oestradiol (27). Oestrogen receptors are overexpressed in pM*Φ* from endometriosis patients (96). In addition, oestrogen levels fluctuate across the menstrual cycle and are also affected by frequently used hormonal treatments, such as the combined contraceptive pill, progestogens or gonadotrophin-releasing hormone agonists and antagonists (2). Mismatches of the hormonal status may impair the reliability and reproducibility of the characterisations. Standardised protocols to collect clinical data and biological samples as well as their processing are the prerequisite for replicable studies and data validation. For endometriosis, these protocols exist and should be used (97–100).

Identification of non-invasive biomarkers is one of the ultimate goals of sEVs studies which requires research beyond the peritoneal microenvironment. Notably, sEV shuttled miR-22–3p, lncRNA aHIF which are identified as key intracellular communicators in the peritoneal microenvironment are significantly higher in the serum of endometriosis patients (90, 101). sEV packaged-miR-214–3p which were reported to have a protective role on endometriosis fibrosis are decreased in serum of women with endometriosis (93). The physiological and pathophysiological significance of these RNAs remains to be determined in endometriosis, but the finding raises hope that sEVs could be the source of a peripheral blood biomarker for diagnosis and assessing the efficacy of treatments of endometriosis. It is necessary to remember that the majority of sEVs studies in endometriosis are pilot studies, we need confirmation and validation of these results using independent and sufficiently powered studies before any conclusion can been drawn. Future work is also required to examine whether engineered-sEVs could become therapeutic modalities, to constrain lesion development and/or improve the quality of life for many women with endometriosis.
